# A Decision Aid for Women Considering Neoadjuvant Systemic Therapy for Operable Invasive Breast Cancer: Development and Protocol of a Phase II Evaluation Study (ANZ1301 DOMINO)

**DOI:** 10.2196/resprot.5641

**Published:** 2016-05-20

**Authors:** Nicholas Zdenkowski, Phyllis Butow, Elizabeth Hutchings, Charles Douglas, Joseph R Coll, Frances M Boyle

**Affiliations:** ^1^Northern Clinical SchoolFaculty of MedicineUniversity of SydneyNorth SydneyAustralia; ^2^Department of Medical OncologyCalvary Mater NewcastleWaratahAustralia; ^3^School of Medicine and Public HealthFaculty of MedicineUnversity of NewcastleCallaghanAustralia; ^4^School of PsychologyUniversity of SydneySydneyAustralia; ^5^Australia and New Zealand Breast Cancer Trials GroupWaratahAustralia; ^6^Patricia Ritchie Centre for Cancer Care and ResearchNorth SydneyAustralia

**Keywords:** breast neoplasm, decision aid, neoadjuvant, chemotherapy, protocol

## Abstract

**Background:**

Neoadjuvant systemic therapy is offered to selected women with large and/or highly proliferative operable breast cancers. This option adds further complexity to an already complex breast cancer treatment decision tree. Patient decision aids are an established method of increasing patient involvement and knowledge while decreasing decisional conflict. There is currently no decision aid available for women considering neoadjuvant systemic therapy.

**Objective:**

We aimed to develop a decision aid for women diagnosed with operable breast cancer and considered suitable for neoadjuvant systemic therapy, and the protocol for a multicenter pre-post study evaluating the acceptability and feasibility of the decision aid.

**Methods:**

The decision aid was developed through literature review, expert advisory panel, adherence to the International Patient Decision Aid Standards, and iterative review. The protocol for evaluation of the decision aid consists of the following: eligible women will undertake a series of questionnaires prior to and after using the decision aid. The primary endpoint is decision aid acceptability to patients and investigators and the feasibility of use. Secondary endpoints include change in decisional conflict, participant knowledge, and information involvement preference. Feasibility is defined as the proportion of eligible participants who use the decision aid to help inform their treatment decision.

**Results:**

This study has recruited 29 out of a planned 50 participants at four Australian sites. A 12-month recruitment period is expected with a further 12-months follow-up.

**Conclusions:**

The decision aid has the potential to allow patients with operable breast cancer, who have been offered neoadjuvant systemic therapy, decreased decisional conflict, and greater involvement in the decision. If this study finds that an online decision aid is feasible and acceptable, it will be made widely available for routine clinical practice.

**Trial Registration:**

Australian and New Zealand Clinical Trials Registry ACTRN12614001267640; http://www.anzctr.org.au/TrialSearch.aspx?searchTxt=ACTRN12614001267640&isBasic=True (Archived by WebCite at http://www.webcitation.org/6gh7BPZdG)

## Introduction

Neoadjuvant systemic therapy (NAST) has become a routine treatment option for selected women with operable breast cancer, endorsed by international guidelines [1, 2], patients [3], doctors [4], and breast cancer advocates [5]. We estimate that at least 20% of patients with breast cancer might benefit from NAST; however, this rate varies among clinicians [6]. It has the advantages of down-staging some larger tumors from mastectomy to lumpectomy [7], providing prognostic information depending on the degree of tumor response [8], and facilitating translational research for early biomarkers of response [9]. In tumors with higher rates of proliferation such as triple negative and HER2 (human epidermal growth factor receptor 2)-positive, pathological complete response is considered a surrogate outcome for the approval of novel therapies [10]. Additional potential benefits include additional time for surgical decision making, genetic testing, and downstaging of the axilla [11]. Overall survival and disease-free survival are equivalent following either neoadjuvant or adjuvant systemic therapy with appropriate local therapy [12]. Despite these advantages, NAST is not frequently used for women with operable disease, with one Australian study reporting an estimated rate of 2.75% [4], and in the United States 3.8% [13]. Possible reasons for this low rate of NAST use include the need for changes in workflow practices, patient expectation for upfront surgery, patient lack of awareness of NAST, and lack of available clinical trials [6]. Potential disadvantages to NAST include the loss of detailed pathology to guide multidisciplinary management; the (low) potential to delay surgery in patients who do not respond to NAST; and reduced time between surgery and radiotherapy, which may impact on breast reconstruction outcomes [4].

In a series of semistructured interviews conducted by our group, women with breast cancer expressed interest in NAST, for down-staging, prognostication, and to allow additional time to plan surgery [3]. However, they were not able to be as involved as they would like in the decision to receive NAST rather than adjuvant systemic therapy. They reported a lack of information, meaning that they did not feel adequately informed about the options available. They felt that clinicians tended to direct them toward one option, rather than their preference of shared control. This skewed distribution of decisional control was echoed in a survey of 207 Australian and New Zealand breast cancer specialists, where the majority of clinicians directed the decision about whether NAST would be given for operable breast cancer. This study, using an adaptation of the Control Preferences Scale [14], found that no clinicians reported that their patients made the final decision about NAST [15]. This indicates a mismatch between patient wishes and the experience of shared decision making [16], and suggests that strategies are required to better involve patients in the decision about NAST.

Women with early stage breast cancer typically desire involvement [17] and decisional control over their treatment [18]. Those who are at least as involved as they wanted experience better decision-related outcomes including consultation satisfaction, satisfaction with decision making, perception of clinician-shared decision-making skills, and decreased decisional conflict [18]. Being involved in decision making about breast cancer is associated with improved quality of life up to 10 years postdiagnosis [19]. However, it may be particularly difficult to engage women in decisions about NAST due to the complexity of the decision, distress from breast cancer diagnosis, perceived urgency, and an expectation that surgery will be the first treatment offered [3]. Patients may also want to proceed with up-front curative surgery in the hope that chemotherapy, which is seen as toxic and intrusive [20], may be avoided entirely.

Patient decision aids (DA) are an established method to improve the quality of shared decision making. Patient decision aids for treatment decisions have been shown to decrease decisional conflict, increase knowledge about options, improve risk perception, and improve patient-practitioner communication [21]. Decision aids are particularly suited to decisions where the various risks and benefits of the alternative treatment options may be valued differently by different individuals [22]. The choice between NAST and conventional sequencing (surgery followed by chemotherapy) is such a decision. In a systematic review of decision aids for patients with early stage breast cancer, we could not find any reports of a decision aid for NAST [23]. In our Australian survey, 86% of breast cancer specialists expressed interest in using a decision aid for women with operable breast cancer who are offered NAST. Women who were interviewed endorsed the development of a NAST DA and expressed a preference for a tool in print form that was accessible from a trusted source. In this paper, we describe the development of such a DA and the protocol for a study that will evaluate that DA.

## Methods

### Decision Aid Development

A DA (see Multimedia Appendix 1) was developed based on a literature review and then refined in an iterative process by an expert advisory panel comprising medical oncologists, breast surgeons, a psycho-oncologist, consumers, a breast care nurse, and a breast cancer advocacy organization representative according to the systematic process described by Coulter et al [24]. A skilled consumer advocate with personal experience of breast cancer reviewed the decision aid on multiple occasions and provided constructive advice. The structure of the DA was based on the International Patient Decision Aid Standards Collaboration statement, to include a balanced description of adjuvant and neoadjuvant therapy, including advantages and disadvantages, outcome probabilities for each option, graphics, and a values clarification exercise. The DA was then circulated to an additional stakeholder group with similar composition to the first, who had not seen the DA, for further refinement. It was then professionally formatted in portable document format (.pdf) to be downloadable and printable in either color or black and white.

The final DA includes an introduction, brief general information about breast cancer and the treatments used, explanation of the options for the timing of chemotherapy and surgery, the advantages and disadvantages of neoadjuvant and adjuvant therapy, a values clarification exercise, a page for notes, a glossary, and information about where to find additional resources. Using the readability statistics package embedded in Microsoft Word, the decision aid has a grade 10 Flesch-Kincaid readability level. The introduction is necessary for newly diagnosed breast cancer patients to understand basic concepts about treatment modalities because they may not have received other written general information at the time that NAST is being discussed. A diagram represents the options of either chemotherapy followed by surgery or surgery followed by chemotherapy. Radiotherapy (if indicated), HER2-directed therapy (if HER2 positive), and endocrine therapy (if estrogen [ER] and/or progesterone receptor [PR] positive) follow in the flow diagram in Multimedia Appendix 1. The diagram is designed to demonstrate that treatment duration is expected to be similar with either option.

Key components of risk are presented using visual, numeric, and narrative formats with appropriate labeling, tailored to individual tumor characteristics [25]. The likelihood of a pathological complete response is presented according to breast cancer subtype: ER/PR (hormone receptor [HR]) positive, HER2 negative; HR positive, HER2 positive; HR negative, HER2 positive; and HR negative and HER2 negative (triple negative [TNBC]). The probability of remaining alive and free of breast cancer at 5 years is presented, based on whether a pathological complete response was achieved, or not. These estimates are based on a meta-analysis of neoadjuvant clinical trial results reported by von Minckwitz et al [26] and Cortazar et al [8]. A 1000-dot diagram, with each dot representing one patient, illustrates the likelihood of tumor progression (3%) or becoming inoperable (0.3%) on neoadjuvant chemotherapy, based on a case series by Caudle et al [27].

The values clarification exercise in this DA is a diagram with statements about advantages and disadvantages of either option [28]. Patients can nominate how important each factor is to them and then make a mark on a linear analogue scale to show which option they prefer and how strong their view is. Patients are encouraged to discuss their options with friends, family, and other health professionals if they wish. The final decision is made at a follow-up visit with their surgeon or medical oncologist.

**Figure 1:**
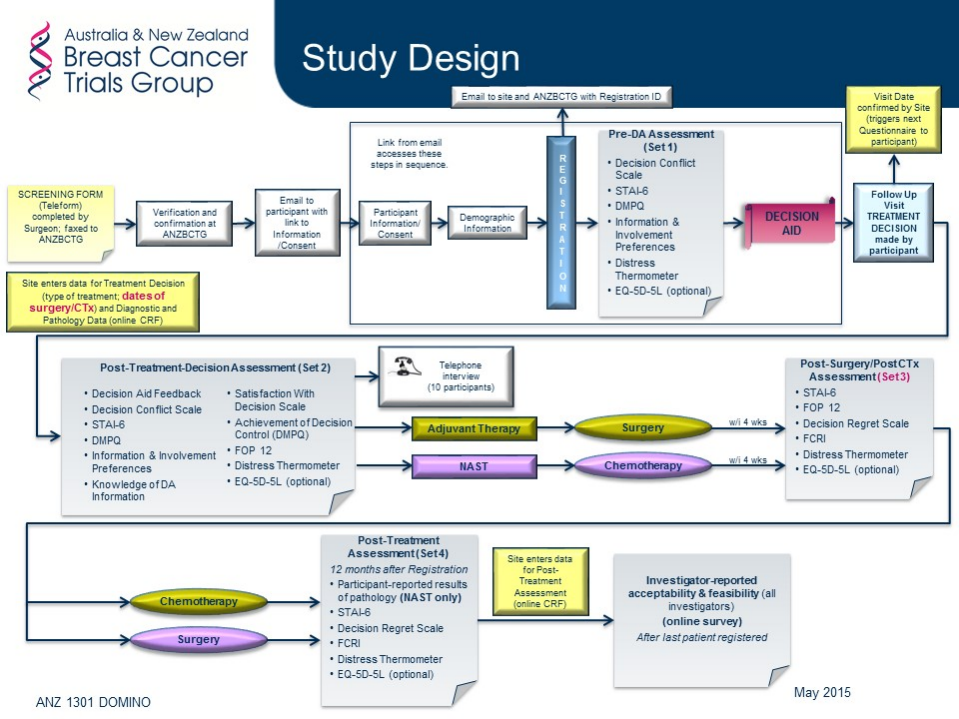
Study schema.

### Evaluation of Decision Aid

ANZ1301 is a multicenter study using a pre-post design to evaluate the acceptability and feasibility of the decision aid (Figure 1) described above. The project is being led by the Australia and New Zealand Breast Cancer Trials Group (ANZBCTG) in collaboration with the Psycho-oncology Co-operative Research Group. It is funded by an HCF Research Foundation grant with central trial coordination by the ANZBCTG. All four sites have received ethics approval from the Hunter New England Local Health District Human Research Ethics Committee, under the Australian National Mutual Acceptance multicenter ethics scheme.

### Study Participants

#### Clinicians

In total, 26 clinicians from four ANZBCTG sites have been recruited to identify women eligible for the DOMINO (DecisiOn MakIng about NeOadjuvant) study. Participating sites are Calvary Mater Newcastle, Waratah, NSW; The Breast and Endocrine Center, Gateshead, NSW; The Mater Hospital, North Sydney, NSW; and Royal Melbourne Hospital, Parkville, VIC. Patients are screened at those four sites and in the private practices of associated clinicians. All participating clinicians and site study personnel receive training on the online system developed for this study.

#### Patients

Patients are eligible to participate in the DOMINO study if they are female and aged over 18 years; have a histological diagnosis of invasive breast cancer; have an operable invasive breast tumour; are considered appropriate for neoadjuvant systemic therapy (NAST) with curative intent using chemo- and/ or endocrine therapy; are able and agree to access study information via the Internet; are able to comply with the study procedures for the duration of the study; and give voluntary, informed consent.

Patients are ineligible if they are expected to receive fewer than 3 months of NAST; have a hearing or other impairment that would preclude a phone interview; are unable to access the Internet using a laptop or desktop computer, or do not have an active email address with which to participate in the study; have insufficient English language skills for participation in online surveys and oral interviews; have inflammatory, metastatic, or inoperable breast cancer; or have a medical or psychiatric condition that precludes informed consent or prevents adherence to study procedures.

### Study Design

#### Screening

Potentially eligible patients are identified during the planning of their initial treatment strategy at participating Australian sites during multidisciplinary meetings and at surgical and medical oncology appointments. Patients are approached by their clinician during their appointment to consider receiving additional information about the DOMINO study via a Web link that is sent to the patient via an auto-generated email. At the time of the initial offer of study participation, patients are asked to indicate written consent on a screening form for their personal information (name, email address, and telephone number) to be provided to the ANZBCTG. A Web link to the study consent page and questionnaires is then sent to them by email. Patients who consent to share personal information are provided with a copy of their signed screening consent form along with study-specific written information about their breast cancer. This information is intended to aid understanding of their diagnosis when using the decision aid. Clinicians are required at this time to record details regarding the primary reason for NAST, as well as an opinion of the patient’s current distress levels and information preferences. The completed screening consent form is sent via Teleform (Hewlett Packard) fax to the ANZBCTG to be verified, and email contact with the patient is established.

If the patient does not consent to share personal information, the clinician records information on the screening consent form so a log can be kept of each patient who is ineligible and screened out, eligible but not offered participation, and offered participation but declines. The number of patients who consent to screening but do not participate in the study will also be recorded. Screening data will be used to describe feasibility of DA use.

#### Registration

Patients are registered to the study through the following process. The screening form is received at the ANZBCTG for validation and confirmation of eligibility criteria. Patients who consent to release their details on the screening form receive an automatically generated email. These patients are now considered eligible for the DOMINO study. Patients who access, read the online DOMINO Information Statement and Consent page, and agree to participate in the study are redirected to a series of demographic questions. On submission of responses to demographics and consent, patients are considered registered to the study. Patients at this stage are also given the opportunity to opt in to a telephone interview. Eligible patients have a 6-week window in which to view and agree to participate in the DOMINO study before being considered a screen failure. We aim to register 50 patients to the DOMINO study.

#### Electronic Communication Processes

At the point of registration, patients are requested to enter a password to access the DOMINO website. Thereafter, an individual’s username is defined by their email address.

Site trial coordinators input dates of patient appointments and projected treatment completion dates, which are then used to calculate and trigger all communication with the patient and reminders to site coordinators. Pre-designed patient and site coordinator emails are sent automatically at specific timepoints, as guided by information entered by sites as to the treatment decision (either NAST or surgery), to patients. A reminder email is sent to the patient if the survey remains unsubmitted 3 calendar days after the initial email. If after an additional 2 calendar days a patient has not submitted a questionnaire, an automatically generated email is sent to the ANZBCTG study coordinator prompting a telephone call to the patient. Site study coordination staff are sent automatically generated emails prompting completion of data via the online system or informing them of their patient’s submission of a questionnaire.

### Outcome Measures

#### Primary Outcomes

DA acceptability is the first outcome. It is defined as at least half of patients considering the DA useful for their decision and at least half of clinicians indicating that they would use the DA in their routine clinical practice. Acceptability will be assessed using a single question from the DA feedback questionnaire developed by Juraskova et al [29] about whether the patient considered the DA useful for their decision. This questionnaire also measures general satisfaction with the DA.

Feasibility of DA use will also be assessed as an outcome. We define it as at least half of patients who were offered participation in the study accessing the DA, and at least half of those who access the DA stating that they read it.

#### Secondary Hypotheses and Outcome Measures

We hypothesize that with use of the DA the Decisional Conflict Scale score will decrease [30]; knowledge about NAST, using a custom-designed questionnaire, will increase; information and involvement preference will increase [31]; agreement between preferred and achieved decision control will be high, based on an adaptation of the Control Preferences Scale to include achieved control [14]; and the Control Preferences Scale score will increase [14]. Further, there will be no change in cost of health care delivery or in the 6-item State-Trait Anxiety Inventory score [32]. Fear of cancer progression will be unchanged while receiving neoadjuvant therapy [33]; the Decisional Regret score after chemotherapy and after surgery will be low [34]; fear of cancer recurrence score will be low [35]; distress thermometer score will decrease [36, 37]; satisfaction with decision score will be high [38]; and there will be no change in outcomes between those who decided to receive neoadjuvant chemotherapy with those who decided not to have neoadjuvant therapy.

#### Exploratory Hypotheses

Correlation will be good between baseline investigator assessment of participant information and involvement preference and participant report of DA acceptability. Correlation will be good between high baseline participant information and involvement preference and high acceptability of DA to participants. Correlation will be good between baseline investigator assessment of distress and participant report of distress. Participants will be willing to complete the EuroQol 5-Dimensions (EQ-5D-5L) questionnaire, a health utility measure.

#### Knowledge Questionnaire

A custom-designed 7-item knowledge questionnaire has been developed based on the content of the DA to test recall and comprehension (see Multimedia Appendix 2). Questions were taken from information throughout the DA. The number of correct responses will be transformed to a score out of 100.

#### Demographic, Tumor, and Treatment Information

The following demographics will be recorded: age, marital status, level of education, health insurance, occupation, and native language. Tumor characteristics consist of tumor size, nodal status, estrogen/progesterone receptor, HER2 amplification, and grade. Investigations and treatment received include duration of chemotherapy, radiotherapy, surgical procedure(s) performed, biopsies, and imaging performed.

#### Telephone Interview

Participants will be asked to participate in a semistructured interview using a pre-planned interview guide, to gain a deeper understanding of their attitude toward the utility and acceptability of the DA. Participants are asked at the time of consent to tick a box indicating their willingness to be contacted for an interview. Interviews will undergo immediate initial analysis and will be conducted until thematic saturation is reached, defined as no new major themes in three consecutive interviews. Further rounds of analysis will be conducted in an iterative fashion after all interviews are complete. Interviews will be recorded, transcribed verbatim, and analyzed using qualitative methodology. Qualitative descriptive methodology will be used, as is appropriate when lived experience, views, and preferences are the target of investigation, and there are little existing data available. This method can be used to gain a rich description of an experience, founded in existing knowledge and interpreted in the context of the clinical experience of the research group [39].

#### Clinician Questionnaire

After 50 patients have completed their post-DA questionnaire, all clinicians will receive an electronic questionnaire. The questions include specialty (surgeon or medical oncologist), intent to use the DA in routine clinical practice, patient selection for DA use, effect on consultation duration and number, apparent effect on decision making, and comments on content. The DA will be considered acceptable to clinicians if more than 50% report that they would use the DA in routine clinical practice.

#### Questionnaire Administration

A series of validated questionnaires where available, and custom designed where a questionnaire is not available, are presented to patients at four timepoints before and after access to the decision aid (see Table 1). Prior to access to the decision aid, patients are asked to report demographics and to complete 6 questionnaires that address decisional conflict, decision-making preference, information and involvement preferences, anxiety, distress, and an optional health economic instrument.

At the completion and submission of the first set of questionnaires, patients are able to access an electronic copy of the decision aid. This document can be printed, saved, and accessed by a patient at any stage of the study by logging in to the DOMINO website. Patients are asked to complete and read the decision aid prior to attending their next appointment with their clinician, at which time a decision regarding treatment may be made. At this visit, the clinician refers to the decision aid and asks whether the patient has any questions about it. After the attendance at an appointment where a decision was made regarding treatment and specific data have been entered by the site, the patient receives an autogenerated email informing them that a second set of questionnaires is available for completion. Questionnaires at this timepoint ask the patient to reflect on the information provided in the DA and its role in their treatment decision. Patients who do not submit both questionnaire sets 1 and 2 will be replaced to ensure that pre-post outcomes are recorded for 50 patients.

Based on treatment details supplied by the site trial coordinator about treatment option chosen and date of completion, an email link to questionnaire set 3 is sent to the patient. This questionnaire is to be completed after the initial treatment strategy of either chemotherapy or surgery. It is expected that most participants will then proceed with surgery or systemic therapy respectively as their subsequent treatment strategy. This assessment aims to determine the effect of the first treatment strategy on decision-related outcomes, without the influence of the alternative strategy.

Questionnaire set 4 is answered 12 months after registration, to investigate longer-term outcomes including anxiety, distress, regret, and recollection of pathology results. This is the last questionnaire, and participants complete their study involvement at this time.

**Table 1:** Questionnaire content according to assessment timepoint.

		Pre-DA assessment	Posttreatment decision assessment	Postchemo assessment (NAST)^a^	Postsurgery (non-NAST)^b^	Posttreatment assessment^c^
Decision conflict scale		X	X			
State-Trait Anxiety Inventory‒6 Anxiety		X	X	X	X	X
**Decision-making preference questionnaire**						
	Preferred	X	X			
	Actual		X			
Distress thermometer		X	X	X	X	X
Information and involvement preferences		X	X			
EQ-5D-5L (optional)		X	X	X	X	X
Knowledge of decision aid information			X			
Decision aid feedback			X			
Satisfaction with decision scale			X			
Fear of progression (FOP 12)			X	X	X	
Decision regret scale				X	X	X
Fear of Cancer Recurrence Inventory				X	X	X
Participant-reported pathology results (NAST only)						X

^a^Postchemotherapy, before surgery.

^b^Postsurgery, before adjuvant chemotherapy.

^c^12 months (+/- 1 month) after registration.

### Statistical Analysis

A sample size of 50 participants is planned. The primary analysis will include all registered patients and clinicians as two separate cohorts. The proportion of patients and investigators who consider the DA acceptable will be reported with 95% exact confidence limits. The primary outcome will be considered positive if more than half of patients and clinicians consider the DA acceptable, and feasible if more than half of eligible patients who are offered participation register and subsequently use the DA. Assuming a sample size of 50 participants, the primary outcome of percentage of participants finding the DA acceptable can be estimated to within ±15% based on 95% exact confidence limits. To ensure that the lower 95% one-tailed exact confidence limit is greater than 50%, at least 32 of the 50 participants will need to indicate DA acceptability. Although the study is not powered to test the secondary hypotheses, there is 80% power to detect a change of at least 0.40 standard deviations from the pre- to post-DA assessments using a two-tailed *t*-test with alpha=.05 and a sample size of 50 participants.

Changes in secondary outcome measures from the pre-DA assessment, including decisional conflict, information preference, anxiety, distress, and fear of progression, will be evaluated using repeated measures analysis of covariance (ANCOVA). All outcomes will be described using mean and standard deviation for continuous measures and frequency for categorical outcomes. If data are skewed, median and interquartile range will be reported and the appropriate linearizing transformation will be used. Analyses will be performed unadjusted and adjusted for age, level of education, information preferences, and tumor characteristics (size, grade, node involvement, ER/PR/HER2). Agreement in decisional control before and after using the DA, and between preferred and actual control, will be assessed using a weighted kappa statistic with McNemar test. Knowledge will be reported as mean proportion of items correct with standard deviation. Cost will be recorded using Australian Medical Benefits Scheme item numbers and Pharmaceutical Benefits Scheme prices, and a comparison made between those who receive surgery first and those who receive systemic therapy first.

#### Missing Data

Patients are encouraged to complete all questions but are not compelled to enter responses to any of the individual questions within each set of questionnaires and can submit responses with blank fields. Prior to questionnaire submission, patients will receive a prompt informing them that not all questions have been answered and to amend if they wish. During the study, levels of missing data are being monitored. If data completion rates drop below 70%, remedial action will be taken. An analysis of missing data will be completed at the end of the study.

## Results

The study is currently recruiting at four Australian centers. As of February 2016, 29 of the planned 50 participants have been registered to the study. Recruitment is expected to be complete in mid-2016, with early results available late 2016.

## Discussion

### Principal Considerations

This study intends to evaluate the acceptability and feasibility of a DA for women with operable breast cancer who have been offered NAST. It is designed as a single arm pre-post study to allow all participants access to the intervention. The population of Australian women who currently receives NAST is relatively small, limiting the feasibility of a larger, randomized controlled trial with comparative outcomes. However, the proportion of patients receiving NAST in Australia and New Zealand is expected to increase as a result of increased awareness, availability of neoadjuvant clinical trials, and from the results of ongoing neoadjuvant and post-neoadjuvant trials.

The study primary endpoints are pragmatic. We expect that some participants will not find the DA beneficial, based on their decision-making style and information-seeking behavior. However, we hypothesize that the number who find it helpful in their decision-making process will be greater than the number who do not find it useful. Because DAs have variable use across centers and individual clinicians [40], feasibility was included as an endpoint. A screening log is designed to quantify the number of patients who are seen at recruiting sites who are candidates for NAST for operable breast cancer; are eligible for the study; are offered study participation; accept study participation; and go on to access the DA. This will identify the proportion of eligible patients who are not offered participation (clinician feasibility) and the proportion of eligible patients who do not access the DA after being offered it (patient feasibility). Acceptability will be assessed using direct questions to patients and clinicians.

Outcome measures were selected based on the availability of valid, reliable questionnaires that assess outcomes relevant to decision making in the context of a decision aid. In a systematic review of the quality of measures to test the effectiveness of decision support strategies, the Decisional Conflict Scale and the Control Preferences Scale satisfied the largest number of quality criteria [41]. These are commonly used measures of DA effectiveness [21, 42]. Knowledge assessment necessitates a custom-designed questionnaire. The Fear of Progression questionnaire is suited to the neoadjuvant setting where the primary cancer is present and has the potential to impact on psychological and physical domains [33]. The Information and Involvement Preferences questionnaire was included to determine the information needs of patients and to correlate the result with patient and clinician acceptability. The EQ-5D-5L is a health economic utility measure [43] and was included as an optional measure to determine patients’ willingness to complete this additional questionnaire. If patients are willing to complete it, then it will be considered for future comparative studies as a health economic measure. Satisfaction with decision is of interest as an acute measure to be assessed after the decision has been made, but prior to experiencing the consequences of the decision [34]. Decision regret is a longer-term outcome measure, to be assessed after the consequences of the decision have been experienced [38].

Increased anxiety is associated with both more effective decision strategies and stressful health interventions, so is not a good measure of the benefit of DA use [44]. Anxiety therefore is not expected to decrease as a result of a DA, but nor should it increase and therefore anxiety has been included as a safety measure [21].

Decision aids are most beneficial if endorsed by a clinician at the time they are offered to the patient and referred to after the decision has been made [45]. This decision aid is introduced at a time when patients have recently been diagnosed with breast cancer and are faced with a number of complex decisions that are influenced by a variety of sources including clinicians, family, and the media [46, 47]. Patients are identified as suitable for the decision aid by their doctor (usually a surgeon) and are generally referred to a medical oncologist to discuss systemic therapy. The decision aid is suited to this situation as there is often a period of several days (or more) before an available appointment with a medical oncologist. If the decision aid is effective, the patient will be more prepared to be involved in the decision when they come to the medical oncologist. Balancing the provision of complex information with adequate readability proved difficult, as demonstrated by a higher Flesch-Kincaid grade than would be ideal.

Decision aids have been shown to have a variable effect on treatment choices [21]. For selected treatment decisions, some patients change their preferred treatment after a DA, but for others decision aids have been shown to have a neutral effect. We anticipate that study patients will not change their decisions after accessing the DA, as decisions such as this tend to be made based on a number of information sources [48].

If shown to be feasible and acceptable, the DOMINO decision aid has the potential to be offered to patients as part of routine clinical practice. There is good evidence for the efficacy of decision aids that are designed according to international standards [22,24]. Thus, a randomized controlled trial is not considered a prerequisite for dissemination. Clinicians who enroll participants in this study will be asked whether they would continue to use the decision aid as part of routine practice, as an indicator of perceived implementability.

### Conclusion

Use of the DOMINO decision aid has the potential to decrease decisional conflict, increase knowledge, and increase patient involvement in women who have been offered NAST. Increased involvement in decisions by women with breast cancer is associated with improved decision-related outcomes [18] and long-term quality of life [19]. Neoadjuvant clinical trials are an established drug development pathway, and the decision aid may allow better understanding of the rationale behind neoadjuvant therapy. The patient may then be able to be better informed about the trial. It may also assist clinicians who are introducing neoadjuvant systemic therapy into their practice but have not yet become confident addressing the concept with their patients.

## Multimedia Appendix 1

Decision aid for neoadjuvant systemic therapy.

## Multimedia Appendix 2

Custom designed knowledge questionnaire.

## Abbreviations

DA: decision aid
ER: estrogen receptor
EQ-5D-5L: EuroQol 5-dimension, 5-level health status measure
HER2: human epidermal growth receptor 2
NAST: neoadjuvant systemic therapy
PR: progesterone receptor
