# Supplementary material for: A Decision Aid for Women Considering Neoadjuvant Systemic Therapy for Operable Invasive Breast Cancer: Development and Protocol of a Phase II Evaluation Study (ANZ1301 DOMINO)
Source: JMIR Res Protoc. 2016 May 20;5(2):e88. doi: 10.2196/resprot.5641 (PMC4893151; doi:10.2196/resprot.5641)
Supplement: Supplementary file 1 [file resprot_v5i2e88_app1.pdf]

A guide for women who are considering breast cancer treatment with chemotherapy and/or hormonal therapy before surgery

Australia & New Zealand
Breast Cancer
Trials Group

Contents

- Introduction 1
- What type of breast cancer do I have? 2
- What treatments might be given for my breast cancer? 3
  - Surgery 3
  - Radiotherapy 3
  - Chemotherapy 3
  - Targeted therapy 3
  - Hormonal therapy 3
- How soon do I need to have treatment? 4
- What are my options for the timing of chemotherapy and surgery? 4
- Why might I choose to have treatment before surgery? 5
  - Reducing the size of the tumour so you can have breast conserving surgery rather than a mastectomy 6
  - Reducing the size of the cancer to make surgery easier so that less breast tissue needs to be removed 7
  - Planning surgery 7
  - Taking part in a breast cancer clinical trial 7
  - Observing the effect of the chemotherapy 7
  - Chances of the tumour disappearing completely 9
  - Prognosis (How likely is the cancer to return?) 10
  - Some other issues with having therapy before surgery 11
  - What if I can't have surgery? 11
- The pros and cons of adjuvant therapy (surgery first) 13
- Arriving at a treatment decision 16
- Worksheet 17
- What happens now? 18
- Glossary 19
- Further information and support 20
- Notes 21

Introduction

Over the last thirty years there have been significant improvements in the treatments available to women diagnosed with breast cancer. More treatment options are available to help cure more women of their cancer, and to keep women alive and well for longer. Treatment has also become more complicated, may be offered at different times and may continue for many months. Whenever possible, your treatment will be planned to best fit your personal situation and preference. Your treatment team is here to help you through this difficult time and to answer questions that you may have. This booklet is designed to help with some of those questions.

In certain situations, doctors may offer treatment with chemotherapy or **hormonal (systemic) therapy *before* surgery.** You have been given this booklet, called a 'Decision Aid', because your doctor thinks that this option may be suitable for you.

The purpose of this decision aid is to help women to choose whether to have chemotherapy or hormonal therapy ***before*** surgery (neoadjuvant) or ***after*** surgery (adjuvant). After reading the information, you may wish to weigh up reasons for and against having neoadjuvant therapy using this decision aid. You can then discuss your thoughts with your doctor and make a decision.

As well as using this decision aid, you might like to talk to your doctor(s), family and friends and read other information that you may have. You may find resources from the Australia and New Zealand Breast Cancer Trials Group, the Cancer Council, Cancer Australia, the Breast Cancer Network Australia (BCNA) and others to be valuable. These resources will give you extra information about breast cancer that you may find useful to help your decision about whether to have systemic therapy before or after surgery. A resource list is available at the end of this booklet along with space for you to make notes.

To summarise, this decision aid booklet is for women who have recently been diagnosed with breast cancer, for whom chemotherapy or hormonal therapy before surgery (neoadjuvant therapy) might be a treatment option.

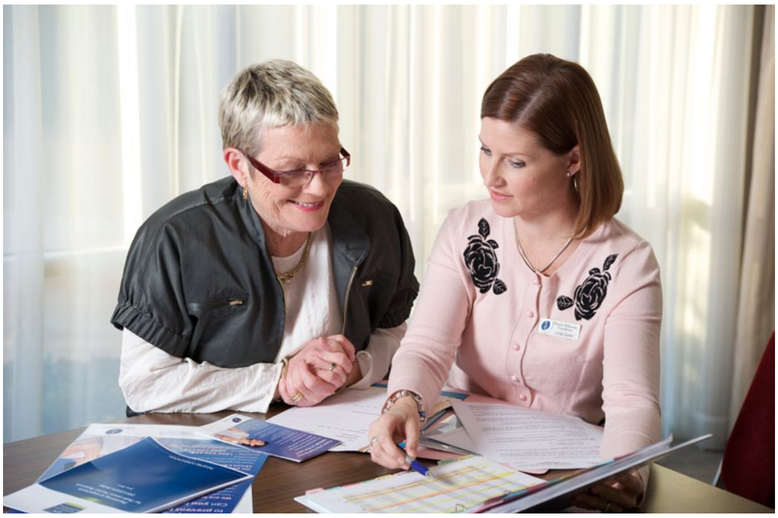

What type of breast cancer do I have?

There are several different types of breast cancer. It is important to know which type you have when thinking about the pros and cons of treatment prior to surgery. Your doctor will be able to give you information about your type of breast cancer. There is also an explanation in the glossary on page 18. You may wish to mark which type you have, as a reminder (just tick the box that describes your cancer).

The main types of breast cancer are:

- ○ Hormone receptor (HR) oestrogen and/or progesterone positive (ER+/PR+), HER2 negative (HER2-)
- ○ Hormone receptor (HR) oestrogen and/or progesterone positive (ER+/PR+), HER2 positive (HER2+)
- ○ Hormone receptor (HR) oestrogen and progesterone negative (ER-/PR-), HER2 positive (HER2+)
- ○ Hormone receptor (HR) oestrogen and progesterone negative (ER-/PR-), HER2 negative (HER2-)(triple negative)

Women with any of these types of cancer may be offered chemotherapy, surgery and radiotherapy. If you have hormone receptor positive breast cancer, you may also be offered hormone blocking medication such as tamoxifen, anastrozole (Arimidex®) or letrozole (Femara®). Similarly, if you have HER2 positive breast cancer, you may be offered a drug called trastuzumab (Herceptin®).

Your doctor may take into account other factors when offering treatment options, including the grade of the tumour (how much tumour cells look like normal cells), stage of the tumour (describes the size and location of cancer), and whether there are cancer cells in the lymph nodes under your arm.

You can make notes about the important features of your cancer here:

What treatments might be given for my breast cancer?

Your doctors and nurses will explain the details about the exact treatments that you could receive. There are usually two types of breast cancer treatment: Local and Systemic.

LOCAL TREATMENTS: Treat cancer cells in the breast area only

Surgery

Surgery involves removing any visible cancer. The whole breast can be removed (a mastectomy) or just the cancer and the area around it (a lumpectomy, or breast conserving surgery). You would also have a procedure to check whether the lymph nodes (glands) in your armpit have any cancer in them. This may be done as a sentinel node biopsy at the same time as your breast operation. If there is cancer in the lymph node(s) that were removed, you may also have an operation to remove some more lymph nodes from under the arm (axillary dissection). Sometimes a second operation is needed because some cancer has been left behind in the breast. Having chemotherapy before surgery does not make this more or less likely to occur.

Radiotherapy

If you have breast conserving surgery, then you are likely to be offered radiotherapy. You may also be offered radiotherapy after a mastectomy, depending on the size of the cancer, whether any lymph nodes are involved, or other factors that your doctors think are important. Radiotherapy kills cancer cells in the area it is aimed at and is similar to the rays that are used when you have a chest X-ray.

SYSTEMIC TREATMENTS: Using drugs that can reach all parts of the body

Chemotherapy

Chemotherapy is a medicine that is given to kill cancer cells throughout the body. For breast cancer, it is usually given intravenously (though a drip or injection into the vein) every 1-3 weeks, for a total of 12-24 weeks. There are many different types of chemotherapy, and your doctor will be able to describe the risks and benefits of the treatments that are most suitable for you.

Targeted therapy

Targeted therapy, such as trastuzumab (Herceptin®), is given to women with HER2 positive breast cancer. This type of treatment works by targeting the HER2 receptors on the tumour cells, stopping the cells from dividing and growing. They are usually given intravenously (through an IV drip) once every three weeks for a year in total, including any trastuzumab that you might receive before surgery.

Hormonal therapy

Hormonal (endocrine) therapy is a tablet that is taken every day, for women with oestrogen (ER) and/or progesterone (PR) receptor positive breast cancer. It works by interfering with the signal that oestrogen sends to cause this type of breast cancer to grow. Hormonal treatments are usually given for 5 years or longer.

Why might it be necessary to have chemotherapy or hormonal therapy?

Chemotherapy and hormonal therapy are given to some women with early breast cancer, to reduce the chance that the cancer will return in the future.

How soon do I need to have treatment?

You might think that treatment for breast cancer needs to start within a very short time. However, research has shown that it takes several years for the majority of breast cancers to grow to a size that can be detected on mammogram. Therefore in most situations there is no harm in taking up to several weeks to make a decision about which treatment is right for you. After you have made a decision, surgery or chemotherapy may take days to a couple of weeks to commence, depending on local scheduling at the hospital where you are planning to have treatment. Again, there is no evidence to suggest that waiting those few weeks makes any difference to the success of your treatment.

What are my options for the timing of chemotherapy and surgery?

Neoadjuvant: This means you will start with chemotherapy, targeted therapy or hormonal therapy, and then have surgery, usually after several months of treatment. Hormonal therapy or targeted therapy (such as trastuzumab) may also continue after surgery. You may also require radiotherapy, which is usually given after surgery. This option does not usually mean that you will receive more therapy or receive treatment for a longer time, just that the timing is different.

Adjuvant: This means you will have surgery first. You will then be offered further therapy, including chemotherapy, targeted therapy, hormonal therapy and radiotherapy depending on the stage and type of cancer you have.

Figure 1. Your options for the sequence of treatments

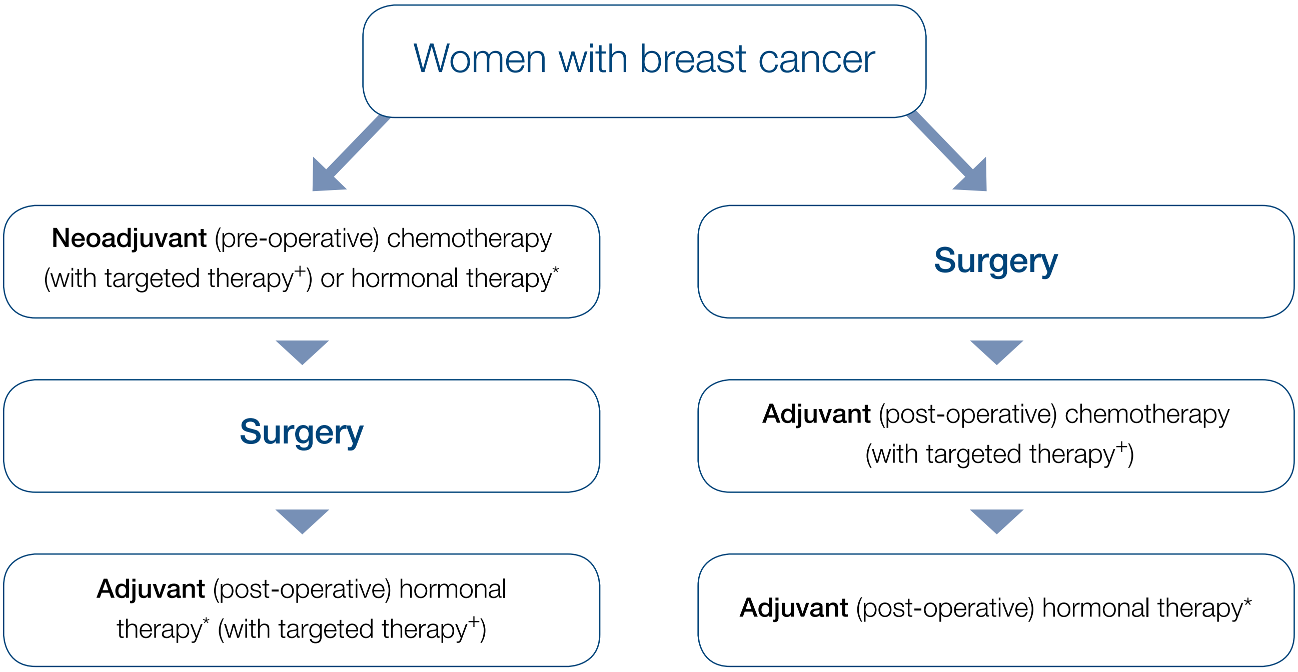

\+ Targeted therapy eg. trastuzumab is for HER2 positive breast cancer

* Hormonal therapy is for hormone receptor positive breast cancer

Neoadjuvant therapy (treatment begins before surgery) has been commonly used around the world for the last 15 years, and is increasingly used in Australia. Breast cancer clinical trials have shown that chemotherapy given to women before surgery is just as effective as having chemotherapy after surgery in terms of the cancer coming back (recurrence), and survival.

Being offered neoadjuvant therapy does not mean that your cancer is worse than cancer treated with adjuvant therapy. Neoadjuvant therapy has in the past been given to women who have larger breast tumours than average, but more recently this type of treatment has been given to women with moderately sized tumours.

Regardless of which treatment you choose, your doctor will ensure that you receive the best possible care.

NEXT, we describe the pros and cons of neoadjuvant therapy, and then the pros and cons of adjuvant therapy.

Why might I choose to have treatment before surgery?

There are several reasons why your doctor might have raised the possibility of treating your cancer with chemotherapy and/or hormonal therapy before it is removed surgically. These reasons include:

- To reduce the size of the tumour so that you can have a lumpectomy (rather than a mastectomy - removing the whole breast);
- To reduce the size of the tumour so that a smaller operation is possible;
- To be eligible to participate in a neoadjuvant clinical trial;
- To give time for more information to become available, such as the results of genetic testing, which can influence the type of surgery and treatment you may choose to have;
- To be able to see or feel the effectiveness of chemotherapy on the cancer;
- To give you a better idea of your prognosis (the chance of your cancer coming back).

These reasons are explained in the following pages. There are other reasons why your doctor may have suggested treatment before surgery, and your doctor will explain these to you, if relevant.

There is space provided for you to make notes at the end of this booklet. If you think of a question or have a concern or comment please write them in the back of the booklet as soon as possible so that you can take this list with you to your next visit.

Reducing the size of the tumour so you can have breast conserving surgery rather than a mastectomy

Your surgeon may have told you that you need a mastectomy (removal of the entire breast). This may be due to the size of the tumour, the type of breast cancer, the size of your breast, or for other reasons.

Neoadjuvant therapy (chemotherapy or hormone therapy before surgery) can reduce the size of the cancer in some patients, so that they may safely have breast conserving surgery (removal of only the part of the breast containing the tumour). The breast cancer is more likely to shrink if it is triple negative or hormone receptor negative/HER2 positive and thus women with these types of breast cancer are more likely to be able to have breast conserving surgery after neoadjuvant therapy.

If you were going to need a mastectomy as initial treatment, but you choose chemotherapy or hormonal therapy before the surgery, you have around a 25% chance of having breast conserving surgery after these treatments. In other words, 25 out of every 100 patients treated in this way will be able to avoid a mastectomy, but 75 in every 100 patients will still require a mastectomy. The diagram below shows this.

Figure 2. If each of the 100 dots below is a woman who was going to need a mastectomy, then after neoadjuvant therapy about 25 women will be able to have breast conserving surgery (empty circles). About 75 women will still need to have a mastectomy (shaded circles).

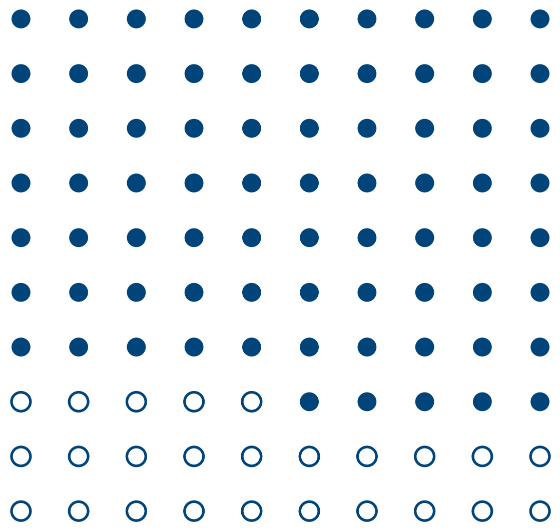

Even if your surgeon has told you that you can have breast conserving surgery now, before chemotherapy is given, then neoadjuvant therapy may shrink the cancer further. This may mean that less breast tissue will need to be taken and your breast is more likely to retain its natural shape.

Reducing the size of the cancer to make surgery easier so that less breast tissue needs to be removed

If scans (ultrasound or MRI) or physical examination show your tumour is more than 2cm in size, then neoadjuvant chemotherapy might shrink the tumour enough so that less breast tissue needs to be removed at the time of surgery.

Planning surgery

In some cases, it may be worthwhile to delay surgery on the breast. You may wish to delay surgery if:

- you are waiting for the results of **genetic testing**. If you are offered genetic testing, and are found to have an inherited breast cancer gene, then you may wish to consider having a double mastectomy (both breasts removed) to reduce your risk of developing breast cancer again. Having the results before surgery means that you can have one operation, rather than two;
- **breast reconstruction** is planned, and you wish to allow time for both your breast cancer surgeon and your reconstruction surgeons to be available to perform their specific surgeries at the same time;
- you wish to delay decisions about surgery, and **take one decision at a time**;
- your doctors feel that it is important to start chemotherapy first because your cancer appears to be fast-growing.

Research has shown that if chemotherapy is required, you will get the same outcomes if you have it before surgery, as having it afterwards. If surgery is delayed for any reason, you might want to get the chemotherapy over and done with and have surgery afterwards.

Taking part in a breast cancer clinical trial

In some situations, neoadjuvant therapy is given as part of a clinical trial (research study). Your doctors will discuss this option with you if there is a trial available that is suitable for you. This may involve a new drug, or a new way of using currently available therapies. For more information about breast cancer clinical trials, visit the Australia and New Zealand Breast Cancer Trials Group website at **www.anzbctg.org** and/or the Australia and New Zealand Clinical Trials Registry at **www.anzctr.org.au.**

Observing the effect of the chemotherapy

If you have chemotherapy or hormonal therapy before surgery, it is possible to check whether the chemotherapy is shrinking your cancer. This occurs for about 90% of women (90 out of every 100 cases treated in this way). **If you have surgery first, there is no way of knowing whether the cancer has shrunk in response to the treatment, because there is no cancer left in the breast to observe.** The reason this is important is discussed below.

Figure 3. Possible results of neoadjuvant chemotherapy.

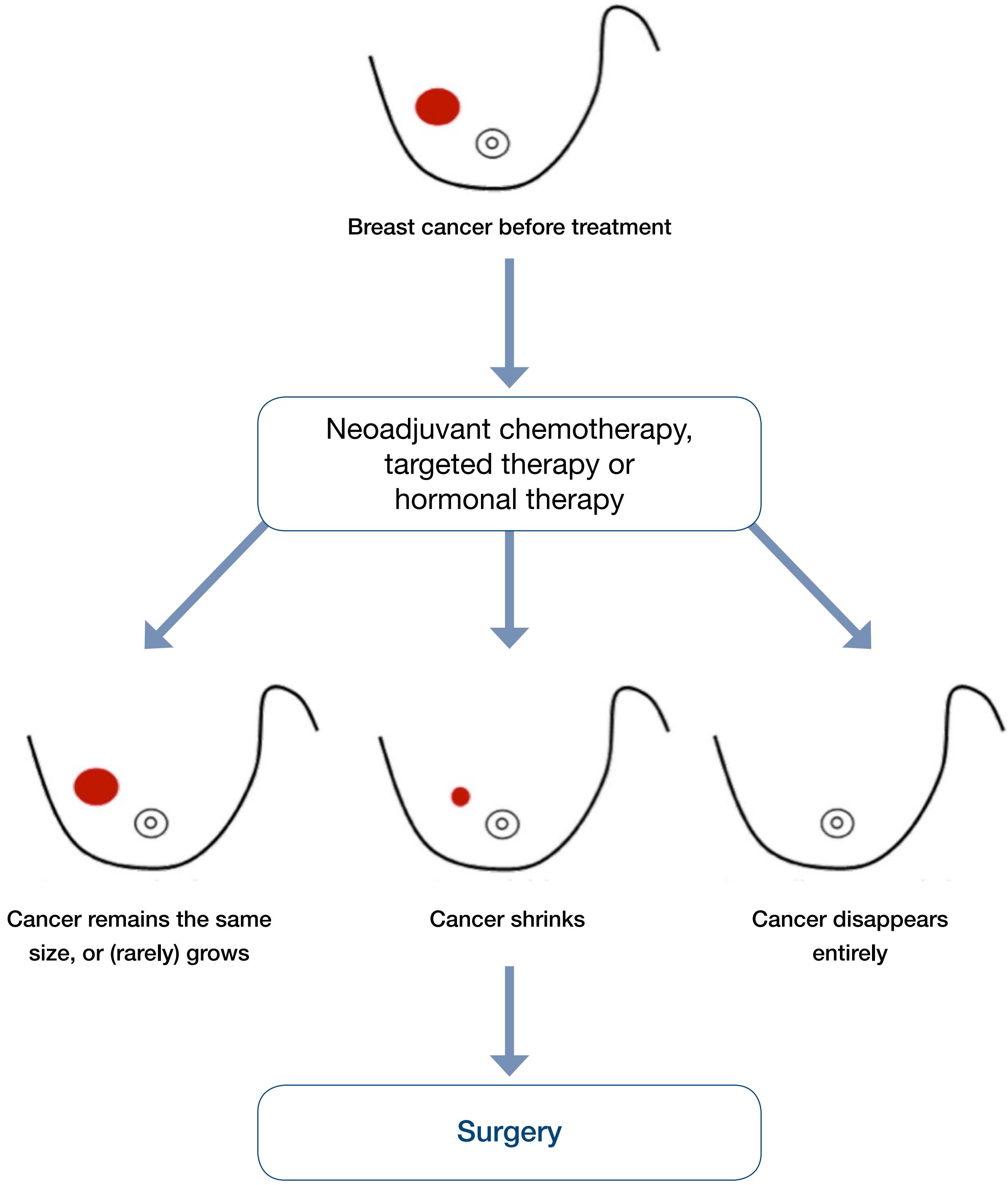

Chances of the tumour disappearing completely

As shown above, there is a chance that neoadjuvant therapy will **completely clear the cancer** from your breast and from the lymph nodes under the arm. In that case, you should still have an operation, as otherwise the cancer may grow back. Your doctor may organise for a special metal ‘clip’ to be injected into the breast cancer before you start or during the course of neoadjuvant chemotherapy so that your surgeon knows where the cancer was and can remove tissue from that area.

The number of patients with **no cancer visible** on surgery after neoadjuvant chemotherapy varies depends on the type of breast cancer:

Figure 4.

Hormone receptor (HR) positive, HER2 negative: 1 out of every 10 women

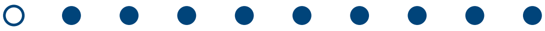

Hormone receptor (HR) positive, HER2 positive: 3 out of every 10 women

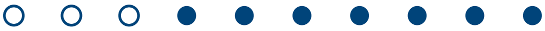

Hormone receptor (HR) negative, HER2 positive: 5 out of every 10 women

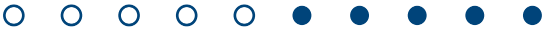

Hormone receptor (HR) negative, HER2 negative (triple negative): 3 out of every 10 women

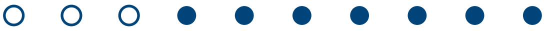

If each of these dots is one woman, the open circles show the number of women (out of 10) who do not have any tumour visible at the end of chemotherapy.

Prognosis (How likely is the cancer to return?)

There is no difference in survival following a diagnosis of breast cancer if you have chemotherapy before or after surgery. If you have chemotherapy or hormonal therapy before surgery, your cancer may shrink. In some cases, the cancer can disappear entirely. Whether or not this happens tells us something about your chances of doing well. If chemotherapy completely clears the cancer from your breast and lymph nodes, you will have gained some "good news" – that your breast cancer is responding to the treatment and is less likely to come back. On the other hand, if your cancer doesn't shrink, or even grows, you will have gained some less good or even "bad" news – that your breast cancer is not so responsive and is *more* likely to appear elsewhere in your body.

The following information is about women who are alive and free of cancer five years after being diagnosed with breast cancer. For some women the cancer comes back before 5 years, for some it comes back after 5 years, and for most women it never comes back. However, the statistics are often described using the 5 year mark.

For women who have had neoadjuvant chemotherapy, the prognosis depends on the type of cancer:

- Hormone receptor positive, HER2 negative: **85 out of every 100** women whose cancer has **disappeared entirely** are alive and free of breast cancer 5 years after diagnosis, compared with **75 out of 100** women whose cancer **did not disappear entirely.**
- Hormone receptor positive, HER2 positive: **90 out of every 100** women whose cancer has **disappeared entirely** are alive and free of breast cancer 5 years after diagnosis, compared with **65 out of 100** women whose cancer **did not disappear entirely.**
- Hormone receptor negative, HER2 positive: **85 out of every 100** women whose cancer has **disappeared entirely** are alive and free of breast cancer 5 years after diagnosis, compared with **50 out of every 100** women whose cancer **did not disappear entirely.**
- Triple negative: **85 out of every 100** women whose cancer has **disappeared entirely** are alive and free of breast cancer 5 years after diagnosis, compared with **50 out of every 100 women** whose cancer **did not disappear entirely.**

The chance of your cancer disappearing completely also depends on which type of cancer you have. This is explained in the following pages.

If the cancer does not shrink or disappear during chemotherapy, it means your cancer is more resistant to the treatment that you were given. It does not mean that the cancer will definitely come back. If you have a hormone positive cancer, not all the work is done by the chemotherapy, hormone blocking treatment is an important part of the treatment of this type of breast cancer.

Even if the cancer cannot be seen on scans and cannot be felt by you or your doctor after neoadjuvant therapy, surgery is still recommended to make sure that all cancer is removed. Looking at the cancer under the microscope after surgery can also give you additional information on the chances that your cancer will return.

Some other issues with having therapy before surgery

There is no increase (or decrease) in problems with surgery

Having chemotherapy and/or hormone therapy before surgery does not increase or decrease the chance of having problems from surgery, such as an infection or delayed wound healing.

Radiotherapy

If you have breast conserving surgery, radiotherapy is usually offered. If you have a mastectomy, radiotherapy may or may not be needed. A good response to neoadjuvant therapy does not mean that radiotherapy can be avoided.

What if the cancer does not get smaller?

As discussed above, there is a chance that the cancer may not seem to be getting any smaller during neoadjuvant therapy. This can cause worry that the treatment is not working. If the cancer does not get any smaller, it does not necessarily mean that the chemotherapy is not working, and you and your doctor may decide to continue with chemotherapy as planned.

In some cases you may still be able to feel a lump in your breast after you have started neoadjuvant therapy. What you can feel or see may only be scar tissue and no cancer is left. On the other hand, even if the cancer can no longer be felt, there is still a possibility that some cancer cells remain. So what we can see and feel from the outside does not tell the whole story. Scans and pathology are needed to make a decision on whether the treatment is working.

What if the cancer gets bigger?

Some people might worry about their cancer getting larger or spreading elsewhere, while receiving treatment prior to surgery. It is uncommon for this to happen. It happens in about 3% (3 in 100) of patients, and in almost all these patients (90%) surgery can still be successfully performed to remove the cancer, with outcomes remaining the same as if they had had surgery before adjuvant treatment. Figure 5 shows this risk.

What if I can't have surgery?

Less than 3 in every 1,000 patients (0.3%) who have neoadjuvant therapy before surgery are not then able to have surgery because the cancer became too large, or because cancer became visible elsewhere in the body (metastatic breast cancer).

In the rare situations that this occurs, it means that surgery would not have cured the cancer anyway, even if an operation was done first. This is because there was already cancer that had spread beyond the breast and lymph nodes, which would not have been completely removed by surgery and chemotherapy.

Your doctor will check regularly with physical examination and scans such as ultrasound or MRI to see whether the cancer is smaller, larger or the same size. If it does grow, your surgery may be moved to an earlier time, before your planned neoadjuvant therapy is finished.

Figure 5. The chance of cancer growing whilst receiving chemotherapy (3%).

Of 1000 people who have neoadjuvant therapy (the blue dots), 30 will have their cancer grow while having treatment (the red dots). 27 of these 30 people are almost certainly still able to have surgery aimed at cure. 3 out of every 1000 (0.3%) would not be able to have surgery, however surgery would not have cured these 3 if done before chemotherapy.

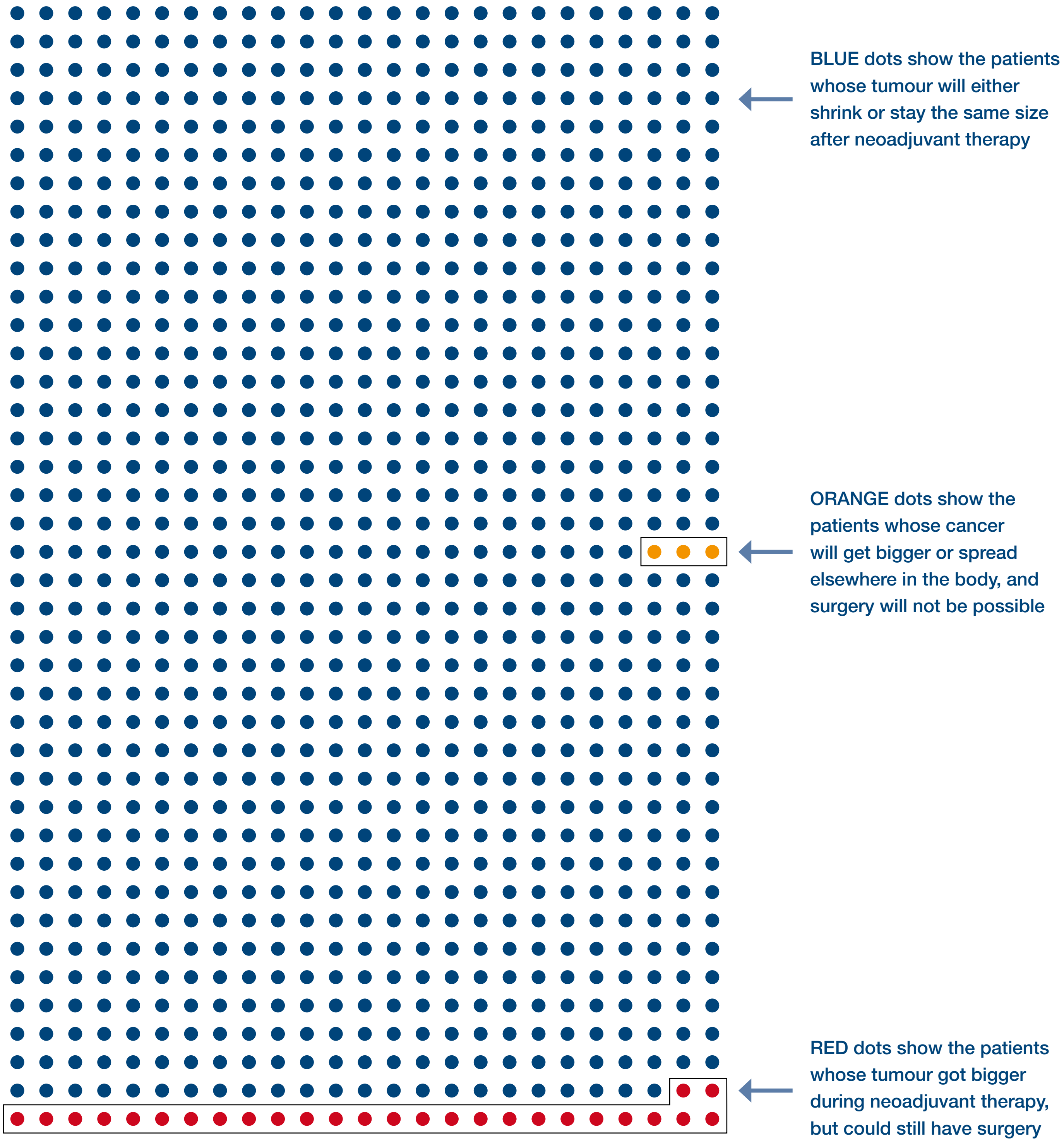

The pros and cons of adjuvant therapy (surgery first)

The chance of the cancer coming back over time is the same if you have chemotherapy first or surgery first.

If an operation is possible now, then you may prefer to have surgery first, and then make a decision about any other treatments such as chemotherapy and radiotherapy, once surgery is complete.

Advantages of surgery first

Information about the cancer

A possible advantage of having surgery first is that you may have more precise knowledge of the type and stage of the cancer before decisions are made about any further treatment. If the cancer is removed in an operation before chemotherapy is given, the pathologist can look at the cancer cells under a microscope and provide information about it. This helps you and your doctors to make decisions about the best treatment options for you. Occasionally this suggests that chemotherapy is not required after all, because the pathologist can see the whole cancer, rather than just a small part of it. The presence of cancer in the lymph nodes near the breast is one of the most useful predictors of prognosis (whether the cancer is likely to return) and it also guides whether radiotherapy is needed. If neoadjuvant chemotherapy (chemotherapy before surgery) has made the cancer shrink or disappear from the breast or lymph nodes, then some of that information may no longer be available.

Immediate removal of the cancer

Some women may be uncomfortable leaving the cancer in place for several months whilst receiving chemotherapy. The idea that the cancer is still in their body can cause some women to feel more anxious and stressed. There is a possibility of anxiety about whether the cancer is growing or not. If the cancer is removed first, then the small chance that it might grow is taken away.

Radiotherapy

If you have a mastectomy, then you are less likely to need radiotherapy after surgery. Radiotherapy might still be needed in certain situations, such as if there is cancer in your lymph nodes under the arm, or if your cancer is large. If you have breast conserving surgery, then radiotherapy is usually offered.

Many women in Australia have surgery first

Having surgery first is common, particularly for small breast cancers where breast conserving surgery is already possible, or in cases where chemotherapy may not be needed. Some women might prefer having a treatment that many others have. The use of neoadjuvant therapy is not rare, but it is less common than having surgery first.

Disadvantages of surgery first

Higher chance of needing a mastectomy

A possible disadvantage of having surgery first is that you may need a mastectomy which could have been avoided if you had neo-adjuvant therapy to shrink the cancer.

No way to see if systemic therapy is working

Another disadvantage is that you will not be able to see the effect that the chemotherapy or hormonal therapy is having on your cancer and so you may have less information about the chance of your cancer coming back. This is discussed in detail in the sections about neoadjuvant therapy titled ‘Observing the effect of chemotherapy’ and ‘Prognosis’.

Infection after surgery

Sometimes after surgery, an infection may develop in the breast, or the wound may be slow to heal, which could delay the start of chemotherapy. While it is usually OK to wait for a short period before starting chemotherapy, it is generally recommended to start within 2-3 months to give the chemotherapy a chance to work. In other words, if an infection causes a delay of more than 2-3 months between surgery and starting chemotherapy, the chemotherapy may be less effective.

Remember:

Being offered treatment prior to surgery does not necessarily mean that there is anything unusual or worse about your type of breast cancer. Neoadjuvant therapy was previously given for larger breast tumours, but more recently this type of treatment is being given to women with moderately sized tumours.

Treatment prior to surgery is commonly used around the world, and is increasingly used in Australia. Having chemotherapy before surgery is just as effective as having chemotherapy after surgery in terms of the chance that the cancer will come back (recurrence) and survival.

The decision to have neoadjuvant or adjuvant therapy is one that you, your surgeon and your medical oncologist can make together. Your doctors will ensure you get the best care regardless of the decision you make.

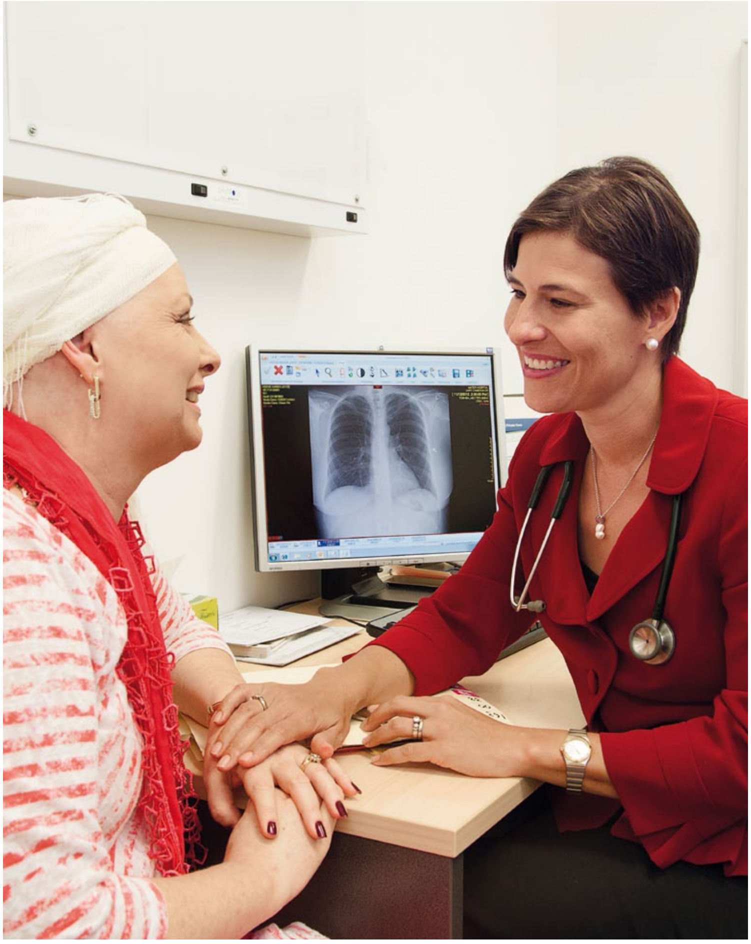

Arriving at a treatment decision

The previous pages have outlined the main options available to you now. The following steps may help you to make a decision whether or not to have chemotherapy or hormonal therapy before surgery.

The decision-making process may be easier if you follow these seven steps:

1. Understand your diagnosis and your risk of breast cancer recurring (coming back) as fully as you can.
2. Understand your options for treatment and the risks and benefits of these options.
3. Review the pros and cons of those options.
4. Assess the importance to *you* of the pros and cons.
5. If you are offered neoadjuvant treatment through a clinical trial, prioritise the pros and cons of the trial for *you* (and your family).
6. Get more information from your doctor or breast care nurse if you are unsure of anything or have more questions.
7. Discuss your preferred treatment option with your surgeon, medical oncologist, family doctor, your family and other significant people in your life.

You have already gone through steps 1-3. To help you complete steps 4-7, and come to the decision that suits you best, we have prepared a worksheet on the following page.

Worksheet

After reading this booklet you may feel you understand more about treatment options for your breast cancer. You may wish to weigh up the positives and negatives, to help you work out which treatment option is right for you. You may not come to a decision now, but this may assist you at your next visit with your medical oncologist or surgeon.

This page lists reasons that are relevant to the decision about whether to receive chemotherapy or hormonal therapy, either before or after surgery for your breast cancer. Indicate which issues are important to you. That will help you work out which way you are leaning.

I want to have breast conserving surgery if at all possible

"I would like to avoid a mastectomy"

Not at all important | Fairly important | Very important

I have reasons to delay surgery

Eg. Genetic testing, planning surgery

Not at all important | Fairly important | Very important

I think it is important to see whether chemotherapy has shrunk the cancer or not

"It may give me information about my prognosis"

Not at all important | Fairly important | Very important

Other

Not at all important | Fairly important | Very important

Favours neoadjuvant (pre-operative) treatment

Waiting for surgery would worry me

"Even if I am having treatment, leaving the tumor there will be stressful"

No Concern | Small Concern | Big Concern

I am worried about the tumor growing or spreading before surgery

"It is rare, but concerning"

No Concern | Small Concern | Big Concern

I want to know as much as possible about the cancer before considering chemotherapy

"It will help my decision about chemotherapy"

No Concern | Small Concern | Big Concern

Other

No Concern | Small Concern | Big Concern

Favours adjuvant (post-operative) treatment

Any further questions? Write down any questions you want to ask your surgeon or medical oncologist (there is more space at the end).

Which way are you leaning? Circle the star which best indicates whether you are leaning towards neoadjuvant therapy or adjuvant therapy. The closer the star is to either option, the more certain you feel about that option.

Which way are you leaning?

Neoadjuvant therapy ★ ★ ★ ★ ★ ★ ★ ★ ★ ★ **Adjuvant therapy**

What happens now?

Your treating doctor, often your surgeon, will have brought up the possibility of neoadjuvant therapy. Your doctor has given you this document to help you find out more about the option of receiving neoadjuvant therapy to assist you to make a decision about whether it is right for you.

Your doctor and other health care professionals (such as a breast care nurse) will continue to support you through the decision-making process, and will be able to answer any questions you might have. You may be referred to see a medical oncologist to discuss the matter further. A referral to a medical oncologist does not mean that you must proceed with treatment before surgery. Neoadjuvant therapy may be recommended as the preferred option, it may be presented to you as a “genuine choice” for you to make, or your medical oncologist may recommend against it, with immediate surgery being the preferred option.

Once you and your doctor(s) have made a decision, treatment will be arranged for you.

Glossary

Adjuvant: treatment that is given after surgery, with the intent of cure

Chemotherapy: Anti-cancer medicine that is usually given through an intravenous (IV) drip

Clinical trial: Research that is testing a new way of treating a disease

Decision aid: a document that contains information to help people make a decision about medical treatment

Early breast cancer: breast cancer that is only in the breast and lymph nodes under the arm on the same side of the body

Endocrine or hormonal therapy: tablets that are taken daily for at least 5 years for hormone receptor positive breast cancer, such as tamoxifen, anastrozole (Arimidex®) or letrozole (Femara®)

HER2 receptor: a protein on the surface of cells that helps them grow and divide

HER2 positive (HER2+) breast cancer: a type of breast cancer that has a larger number of HER2 receptors on the cells than usual. It can be treated with drugs such as trastuzumab (Herceptin®)

Hormone receptor: either the oestrogen receptor or progesterone receptor, which indicates that hormonal treatments such as tamoxifen may be used

Hormone receptor positive breast cancer (ER+/PR+): a type of breast cancer that has hormone receptors on the cells. These receptors are special proteins that the hormones estrogen and progesterone bind to, causing the cancer to grow. Hormonal therapy can be used for this type of breast cancer

Inflammatory breast cancer: breast cancer that affects a large area of the breast, but may not be felt as a distinct lump. It is a less common type of breast cancer; your doctor will be able to tell you if you have this type.

Local therapy: Treatment that affects part of the body, eg. surgery or radiotherapy

Metastatic (secondary or advanced) breast cancer: breast cancer that has spread beyond the breast and lymph nodes, to other parts of the body, such as bones, liver or lungs

Neoadjuvant: treatment that is given before an operation to remove the breast cancer

Pathological complete response (pCR): when no cancer can be seen by the pathologist in the breast or lymph nodes that have been surgically removed, after neoadjuvant chemotherapy or hormonal therapy has been given

Systemic therapy: Treatment that affects the whole body, eg. chemotherapy or hormonal therapy

Triple negative breast cancer: breast cancer that does not have oestrogen (ER), progesterone (PR), or HER2 receptors on its surface

Further information and support

Australia and New Zealand Breast Cancer Trials Group: **www.anzbctg.org**

Australian New Zealand Clinical Trials Registry: **www.anzctr.org.au**

Breast Cancer Network Australia: **www.bcna.org.au**
Phone 1800 500 258

Cancer Australia: **www.canceraustralia.gov.au**
Phone 1800 624 973

Cancer Council **www.cancercouncil.com.au**
Phone 131 120

Macmillan Cancer Support: **www.macmillan.org.uk** (United Kingdom based information)

Local contact information:

Notes

Full title: Decision aid for women considering having chemotherapy or hormonal therapy prior to surgery for breast cancer

This document has been developed by researchers of the Australia and New Zealand Breast Cancer Trials Group (ANZBCTG) and the Patricia Ritchie Centre for Cancer Care and Research, with funding support from the HCF Research Foundation, and the fundraising and education department of the ANZBCTG, the Breast Cancer Institute of Australia (BCIA). It has been endorsed by the ANZBCTG Consumer Advisory Panel. The Breast Cancer Network Australia (BCNA) have provided input into content.

1st edition, December 2014.

© Australia and New Zealand Breast Cancer Trials Group

Australia & New Zealand Breast Cancer Trials Group

Breast Cancer Network Australia
