# Supplementary material for: A Decision Aid for Women Considering Neoadjuvant Systemic Therapy for Operable Invasive Breast Cancer: Development and Protocol of a Phase II Evaluation Study (ANZ1301 DOMINO)
Source: JMIR Res Protoc. 2016 May 20;5(2):e88. doi: 10.2196/resprot.5641 (PMC4893151; doi:10.2196/resprot.5641)
Supplement: Supplementary file 2 [file resprot_v5i2e88_app2.pdf]

Knowledge of DA Information

ALL ASSESSMENTS WILL BE COMPLETED ONLINE.

NO PAPER COPIES ARE TO BE PRINTED AND GIVEN TO TRIAL PARTICIPANTS

Please read each of these statements and place a mark in the box to show whether you believe it to be true, false, or you are unsure:

	Definitely true	Probably true	I am unsure	Probably false	Definitely false
Neoadjuvant treatment involves receiving treatment <u>before</u> surgery for breast cancer	☐	☐	☐	☐	☐
Patients who receive neoadjuvant treatment have the same chance of the cancer coming back as those who have surgery first	☐	☐	☐	☐	☐
There is a chance that my cancer will disappear completely	☐	☐	☐	☐	☐
If my cancer disappears entirely, I may not need surgery	☐	☐	☐	☐	☐
If the cancer disappears entirely, it is less likely to come back	☐	☐	☐	☐	☐
If my cancer shrinks after neoadjuvant therapy, I will still have to have the same type of surgery (e.g. mastectomy)	☐	☐	☐	☐	☐
The chance of the cancer getting smaller depends on the type of breast cancer that it is	☐	☐	☐	☐	☐
